# Prognostic factors and nomogram for cancer-specific death in non small cell lung cancer with malignant pericardial effusion

**DOI:** 10.1371/journal.pone.0217007

**Published:** 2019-05-16

**Authors:** Zhi Gang Hu, Ke Hu, Wen Xin Li, Fan Jun Zeng

**Affiliations:** 1 Department of Respiratory Medicine, Renmin Hospital of Wuhan University, Wuhan, Hubei Province, People’s Republic of China; 2 Department of Respiratory medicine, Respiratory Disease Research Institute of China, Three Gorges University, Yichang, Hubei Province, People’s Republic of China; Weill Cornell Medical College in Qatar, QATAR

## Abstract

**Background:**

The prognosis of lung cancer with malignant pericardial effusion is very terrible owing to the impact of cardiac tamponade. The aim of our study seeks to identify prognostic factors and establish a prognostic nomogram of non small cell lung cancer (NSCLC) with malignant pericardial effusion.

**Methods:**

NSCLC patients with malignant pericardial effusion between 2010 and 2014 are searched from SEER database.Cancer-specific death of these patients are analyzed through the Kaplan–Meier method, Cox proportional hazard model and competing risk model. Prognostic nomogram of cancer-specific death is performed and validated with concordance index (C-index), calibration plots and internal validation population. Propensity score matching is used to evaluate whether chemotherapy affected the survival of study population.

**Results:**

696 eligible NSCLC patients are involved in the study population, with 22.7% of 1-year survival rate and 8.9% of 2-year survival rate. Laterality, AJCC N, AJCC T, and chemotherapy are regarded as independent prognostic factors of cancer-specific death in the Cox proportional hazards model and competing risk model. The C-index of established nomogram is 0.703(95%CI:0.68–0.73) for cancer-specific death in the study population with acceptable calibration, which is significantly higher than classical TNM stage(C-index = 0.56, 95%CI:0.52–0.60). After 1:1 propensity score matching, chemotherapy potentially reduces the risk of cancer-specific death (HR = 0.42 95%CI: 0.31–0.58) of NSCLC with pericardial effusion.

**Conclusions:**

NSCLC with malignant pericardial effusion harbors low overall survival. One prognostic nomogram based on laterality, AJCC N, AJCC T and chemotherapy is developed for cancer-specific death to predict 1-year and 2-year survival rate with good performance.

## Introduction

Pericardial effusion is defined as the pathological accumulation of fluid within the pericardial cavity. Pericardial effusion is often asymptomatic. However, malignant pericardial effusion brings numerous discomforts to the patient through rapid accumulation, including tightness, hyperpnea, dyspnoea and chest pain. The most serious clinical manifestation of malignant pericardial effusion is cardiac tamponade, which may lead to life-threatening complications without prompt treatment. Malignant neoplasms in autopsy series, including lung cancer, breast cancer, lymphma, and esophagogastric cancer, are responsible for 2%–20% of pericardial effusion [1–3]. Compared with non-cancer related pericardial effusion, malignant pericardial effusion has a higher recurrence rate and poorer prognosis [4,5].

More than one-third of malignant pericardial effusion result from lung cancer with high recurrence rate [3,4,6]. According to the UICC seventh TNM edition of lung cancer, malignant pericardial effusion is classified as M1a and leads to stage IV [7]. The median survival time of lung cancer with pericardial effusion is three months or even less, which is significantly lower than those of tumors [3,8,9]. Among the different pathological types of non small cell lung cancer (NSCLC), the incidences of malignant pericardial effusion are approximately 2.2%–3.3%, with 1-year survival rate reaching an average of 14.4%–24.3% [10]. Pericardial effusion is confirmed to be an independent prognostic factor of lung cancer death. Kato and his colleagues [11] found that advanced NSCLC with malignant pericardial effusion had lower median survival time (7.6 months) than that without malignant pericardial effusion (15.0 months). Malignant pericardial effusion significantly increased the risk of lung cancer death when compared with other M1a descriptors (malignant pleural dissemination or contralateral intrapulmonary nodules) [10]. The overall survival of lung cancer patient with pericardial effusion was considerably dismal and similar to that of patients with distant metastasis (M1b). However, only few studies have evaluated the independent prognostic factors of malignant pericardial effusion-related death [3,6,9]. In one study involving 275 patients, clinical presentation of tamponade, positive fluid cytology for malignant cells, lung cancer and male were identified as independent risk factors of pericardial effusion in the multivariate Cox regression analysis [3]. In another study, NSCLC, pleural effusion, and positive cytology were associated with poor prognosis through the multivariate Cox regression analysis [9]. Yonemori and his colleagues [6] found that performance status, mediastinal lymph node metastasis, adenocarcinoma and malignant pericardial effusion during chemotherapy were negatively related to survival time. To date, no large-sample study has been conducted to evaluate the prognostic factor of pericardial effusion with lung cancer alone through the multivariate Cox regression analysis. In addition, some clinicians only recommend percutaneous tube pericadiostomy to relieve the clinical symptoms of the patients with malignant pericardial effusion owing to their short survival time [12,13]. The effects of systemic chemotherapy in treating malignant pericardial effusion secondary to lung cancer are controversial [14–16]. However, no large-sample randomized controlled trial is available to evaluate whether chemotherapy improves the overall survival of lung cancer with pericardial effusion.

In our study, multivariate Cox regression analysis and competing risk analysis were used to identify prognostic factors and develop a prognostic nomogram of cancer-specific death for non small cell lung cancer(NSCLC) with pericardial effusion by analyzing the patient’s available data in the Surveillance, Epidemiology, and End Results (SEER)-Medicare database. The validity of prognostic nomogram was examined by using concordance index (C-index), calibration plots and internal validation. Propensity score matching was deemed more suitable for non-randomized study because of its ability to decrease the potential selection bias [17].Through propensity score matching, we evaluated the impact of chemotherapy on cancer-specific death in NSCLC patients with pericardial effusion.

## Materials and methods

### Data and variable selection

All the data of lung cancer patients with pericardial effusion come from the latest release of SEER-18 registry database between January 2010 and December 2014. The follow-up deadline was November 2016.We focused on this period for two reasons:On the one hand, the deadline of present SEER data was Dec 2014 and provided additional treatment information.On the other hand, the stage of lung cancer was based on the American Joint Committee on Cancer Seven editors staging system since 2010. The eligible patients were searched by using “CS mets at dx = 20 or 21” and “Site recode ICD-0-3/WHO 2008 = Lung and bronchus”.All lung cancer patients were diagnosed through positive histology, positive exfoliative cytology and positive microscope confirm. The following variables were collected:age, sex, primary site, laterality, ICD-O-3 Hist/behav, malignant, Derived AJCC T (7th ed), Derived AJCC N (7th ed), Derived AJCC M(7th ed), Chemotherapy (yes, no/unknown), SEER cause-specific death classification, Survival months, Vital status recode (study cutoff used), Race recode (W, B, other), Marital status at diagnosis(yes,no), Rural-Urban Continuum Code, Lung—Tumor Size, contralateral or bilateral pleural effusion (yes, no).The patients in the study population must include detailed data in the above-mentioned variables. Through the multivariate cox regression analysis and competing risk analysis, some variables may be confirmed as independent prognostic factors of cancer-specific death. Age groups were subdivided into less than 56 years old, 56 to 75 years old, and older than 75 years old. Primary site groups were subdivided into main bronchus and single lobe. Laterality groups were subdivided into left and right.ICD-O-3 Hist groups were subdivided into adenocarcinoma, squamous cell carcinoma and other (malignant neoplasm and carcinoma were discarded). Race recode groups were subdivided into white, black and other. Marital status at diagnosis groups were subdivided into married and unmarried. Rural-Urban Continuum Code groups were subdivided into counties, Urban, comp-rural.Lung tumor size groups were subdivided into less than 36 mm, 36 to 70 mm, and more than 70 mm.

### Ethics

Because all data derived from open SEER database, there were no patients involved in the recruitment and conduct of the study.This study was deemed exempt for review by the Institutional Review Board at Renmin Hospital of Wuhan University.

### Statistical analysis

Median survival times (range) of all variables in cancer-specific death were calculated using Kaplan-Meier method. All variables in the study population were considered in multivariate regression analysis by using the Cox proportional hazard model. The authors assessed the problem of collinearity of all variables through tolerance and variance inflation factor. If the tolerance of variable was less than 0.1 and variance inflation factor was greater than 5, then this variable would be considered for removal from this study. In addition, competing risk analysis was used to test further whether the risk factors were associated with cancer-specific death through the application of proportional subdistribution hazard regression, which could provide a cumulative incidence function to evaluate the unbiased risks of cancer-specific death. Akaike information criterion and the Bayesian information criterion (BIC) were used to competing risk analysis. All reported *P* values were two-sided. If the *P* value of a single variable in the multivariate cox regression analysis and competing risk analysis was less than 0.05, then this variable would be regarded as the independent prognostic factor of cancer-specific death. Hazard ratio (HR) and subdistribution hazard ratio(sbHR) were used to describe the impact of a single variable on the NSCLC patients with pericardial effusion in the cox proportional hazard model and the proportional subdistribution hazard model, respectively. All relevant independent prognostic factors of cancer-specific death were used to construct their prognostic nomogram at 1-year survival and 2-year survival. Concordance index (c-index) and calibration plots were used to evaluate the statistical performance of the prognostic nomogram based on the Cox model.The value of the C-index statistic ranged from 0.5 (no discrimination) to 1 (perfect discrimination), and higher C-index values indicated a better prognostic model.Bootstraps with 500 resamples were used to decrease overfit bias. In addition, the authors also assessed the performance of the prognostic nomogram through internal validation. Finally, we performed a 1:1 propensity score matching to minimize the potential selection bias in the study population, which lead to better evaluate the impact of chemotherapy on cancer-specific death of lung cancer patients with pericardial effusion. Spss 22.0, R software and stata were used to perform the above-mentioned data analyses.

## Results

### Patient characteristics

**Fig 1:**
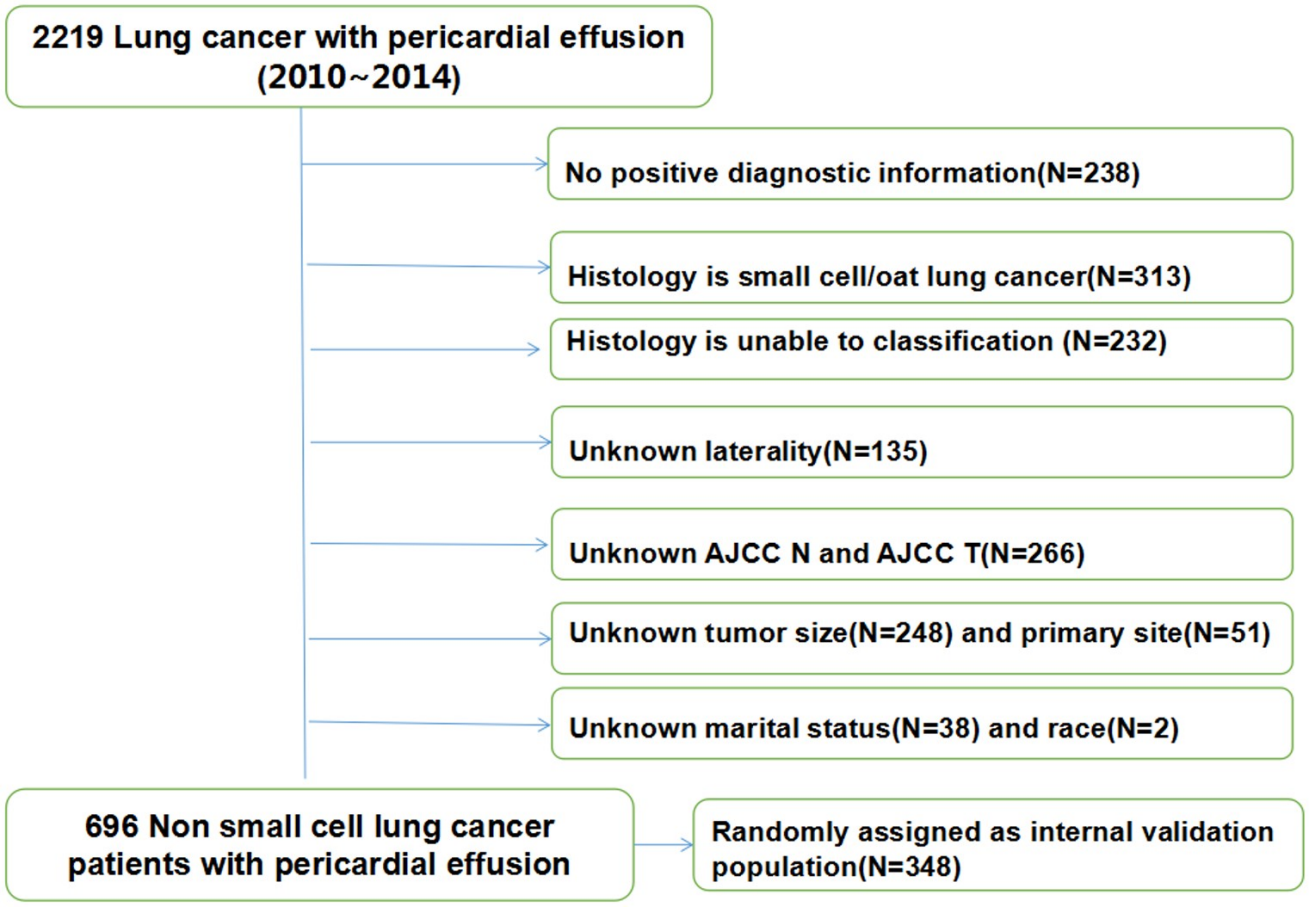
Flow chart detailing the selection of the patients in this study.

A total of 2219 lung cancer patients were associated with malignant pericardial effusion in the SEER database, including 731 patients with contralateral or bilateral pleural effusion.There were 1981 patients with positive diagnostic confirmation. Finally, 696 NSCLC patients were divided into the study population, which included detailed data in the aforementioned variables. The patient selection was summarized in Fig 1.

Clinical characteristics of the study population were listed in Table 1. In the study population, there were 22.7% of 1-year survival rate and 8.9% of 2-year survival rate respectively, with a median follow-up of 8.4 months. During the follow-up period, there were 403 patients of cancer-specific death (57.9%) and 155 patients of other cause-specific death (22.3%).

**Table 1:** Baseline characteristics of non small cell lung cancer patients with malignant pericardial effusion.

Variable	Number	Cancer-specific death	Other cause- specific death	Alive	Median survival time(months)
Sex					
Female	350 (50.3%)	201 (49.9%)	80 (51.6%)	69 (50.0%)	8(6.0 to 10.1)
Male	346 (49.7%)	202 (50.1%)	75 (48.4%)	69 (50.0%)	8(6.1 to 9.9)
Race					
White	537 (77.2%)	309 (76.7%)	129 (83.2%)	99 (71.7%)	8(6.4 to 9.6)
Black	110 (15.8%)	65 (16.1%)	17 (11.0%)	28 (20.3%)	7(4.7 to 9.3)
Other	49 (7.0%)	29 (7.2%)	9 (5.8%)	11 (8.0%)	9(0 to 18)
Marital status					
Married	347 (49.9%)	192 (47.6%)	82 (52.9%)	73 (52.9%)	10(7.7 to 12.3)
Unmarried	349 (50.1%)	211 (52.4%)	73 (47.1%)	65 (47.1%)	7(5.4 to 8.6)
Pleural effusion					
No	484 (69.5%)	275 (68.2%)	106 (68.4%)	103 (74.6%)	9(7.4 to 10.6)
Yes	212 (30.5%)	128 (31.8%)	49 (31.6%)	35 (25.4%)	6(4.0 to 8.0)
Age					
≤50 years	43 (6.2%)	150 (37.2%)	65 (41.9%)	58 (42.0%)	10(5.8 to 14.2)
51 years to 75 years	466 (67.0%)	159 (39.5%)	58 (37.4%)	57 (41.3%)	5(4.0 to 6.0)
> 75 years	187 (26.9%)	94 (23.3%)	32 (20.6%)	23 (16.7%)	3(1.9 to 4.1)
Tumor size					
≤35 mm	273 (39.2%)	26 (6.5%)	6 (3.9%)	11 (8.0%)	11(8.3 to 13.7)
36 mm to 70mm	274 (39.4%)	273 (67.7%)	87 (56.1%)	106 (76.8%)	8(6.2 to 9.8)
> 70 mm	149 (21.4%)	104 (25.8%)	62 (40.0%)	21 (15.2%)	5(3.0 to 7.0)
Primary Site					
Upper lobe	436 (62.6%)	253 (62.8%)	99 (63.9%)	84 (60.9%)	7(5.2 to 8.8)
Middle lobe	36 (5.2%)	24 (6.0%)	4 (2.6%)	8 (5.8%)	9(5.0 to 13.0)
Lower lobe	178 (25.6%)	99 (24.6%)	43 (27.7%)	36 (26.1%)	9(6.3 to 11.7)
Main bronchus	46 (6.6%)	27 (6.7%)	9 (5.8%)	10 (7.2%)	5(1.17 to 8.8)
Laterality					
Left	307 (44.1%)	183 (45.4%)	67 (43.2%)	57 (41.3%)	7(5.2 to 8.8)
Right	389 (55.9%)	220 (54.6%)	88 (56.8%)	81 (58.7%)	9(7.1 to 10.9)
Histology					
Squamous cell carcinoma	234 (33.6%)	130 (32.3%)	68 (43.9%)	36 (26.1%)	7(4.5 to 9.5)
Adenocarcinoma	405 (58.2%)	237 (58.8%)	76 (49.0%)	92 (66.7%)	9(7.0 to 11.0)
Other	57 (8.2%)	36 (8.9%)	11 (7.1%)	10 (7.2%)	8(3.1 to 12.9)
AJCC T, 7th ed					
T1	98 (14.1%)	48 (11.9%)	24 (15.5%)	26 (18.8%)	14(6.0 to 22)
T2	203 (29.2%)	119 (29.5%)	43 (27.7%)	41 (29.7%)	9(6.8 to 11.2)
T3	174 (25.0%)	96 (23.8%)	37 (23.9%)	41 (29.7%)	7(3.9 to 10.1)
T4	221 (31.8%)	140 (34.7%)	51 (32.9%)	30 (21.7%)	6(4.0 to 8.0)
AJCC N, 7th ed					
N0	139 (20.0%)	65 (16.1%)	42 (27.1%)	32 (23.2%)	18(13.9 to 22.1)
N1	42 (6.0%)	20 (5.0%)	8 (5.2%)	14 (10.1%)	11(2.3 to 19.7)
N2	352 (50.6%)	212 (52.6%)	72 (46.5%)	68 (49.3%)	7(5.2 to 8.8)
N3	163 (23.4%)	106 (26.3%)	33 (21.3%)	24 (17.4%)	6(4.2 to 7.8)
Chemotherapy					
No	369 (53.0%)	220 (54.6%)	102 (65.8%)	47 (34.1%)	4(3.0 to 5.0)
Yes	327 (47.0%)	183 (45.4%)	53 (34.2%)	91 (65.9%)	12(9.9 to 14.1)

### Independent prognostic factors in the study population

Kaplan-Meier method was used to calculate the median survival time of all the above-mentioned variables on cancer-specific death (Table 1). All variables were entered into the multivariate cox regression analysis of cancer-specific death with the estimate of collinearity. Laterality, AJCC N, AJCC T, and chemotherapy were regarded as the independent prognostic factors of cancer-specific death (Table 2). Compared with the left-origin of primary lung cancer, the patients with the right-origin of primary lung cancer were associated with longer median survival time (7 months vs 9 months, HR = 0.75, 95%CI: 0.61–0.92, *P = 0*.*01*). AJCC N and AJCC T were positively associated with the cancer-specific death of lung cancer patients with pericardial effusion. The patients with chemotherapy harbored the longer survival time than the patients without chemotherapy (12 months vs 4 months, HR = 0.38, 95%CI: 0.31–0.47, *P*<0.001). In the competing risk analysis, Laterality, AJCC N, AJCC T, and chemotherapy still were considered as independent prognostic factors of cancer-specific death. The detailed sbHR of every variable in the competing risk model were listed in Table 2.

**Table 2:** The independent risk factors of cancer-specific death of non small cell lung cancer and malignant pericardial effusion in the multivariate Cox hazards model and the competing risk model.

	Multivariate Cox analysis^1^		Competing risks analysis^2^	
	HR (95% CI)	*P* value	sbHR (95% CI)	*P* value
Laterality				
Left	1.00		1.00	
Right	0.75(0.61–0.92)	0.01	0.7(0.60–0.96)	0.02
AJCC T, 7th ed				
T1	1.00		1.00	
T2	1.27(1.90–1.78)	0.17	1.23(0.83–1.83)	0.30
T3	1.42(1.0–2.02)	0.05	1.39(0.92–2.09)	0.11
T4	1.66(1.19–2.31)	<0.01	1.67(1.14–2.46)	0.01
AJCC N, 7th ed				
N0	1.00		1.00	
N1	1.61(0.96–2.71)	0.07	1.04(0.53–2.03)	0.91
N2	2.22(1.65–3.0)	<0.01	1.93(1.36–2.73)	<0.01
N3	2.38(1.72–3.30)	<0.01	2.32(1.58–3.39)	<0.01
Chemotherapy				
No	1.00		1.00	
Yes	0.38(0.31–0.47)	<0.01	0.67(0.53–0.86)	<0.01

^1^ Using Cox proportional hazards regression model

^2^ Using proportional subdistribution hazards regression Model

### Nomogram development and validation

**Fig 2:**
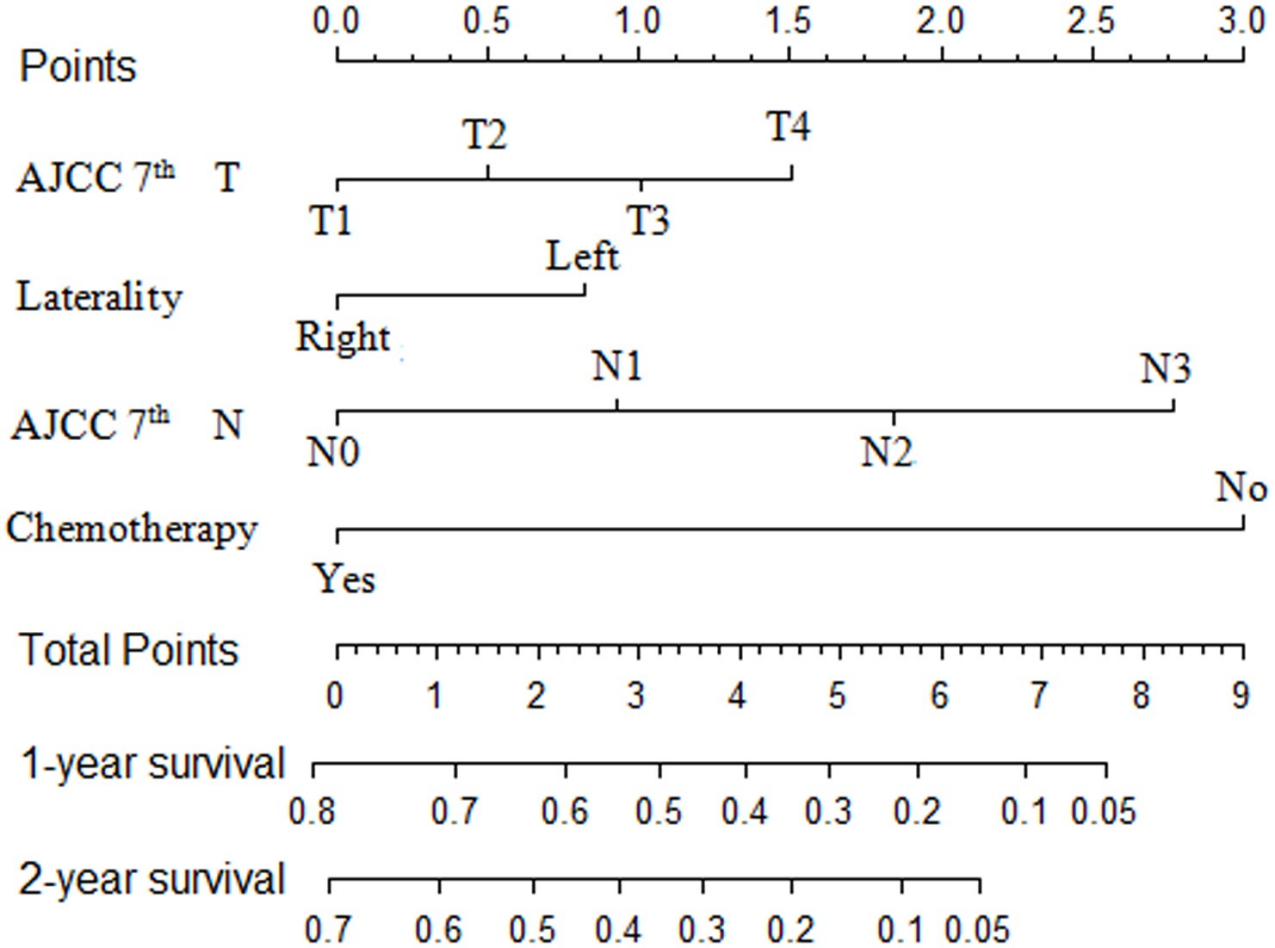
Prognostic nomogram of cause-specific death in lung cancer patients with malignant pericardial effusion.

The prognostic nomogram of cancer-specific death was established to predict 1-year and 2-year survival rate in the study population, based on laterality, AJCC N, AJCC T, and chemotherapy. Each prognostic factor was given a score on the point scale. Clinicians have the ability to estimate 1-year and 2-year survival rate of lung cancer patients with malignant pericardial effusion by determining the score of each prognostic factor and calculating their total score. The prognostic nomogram of cancer-specific death illustrated that chemotherapy shares the largest contribution, followed by AJCC N, AJCC T and laterality (Fig 2).

The validation of prognostic nomogram was evaluated through C-index and calibration plot. The C-index of an established nomogram was 0.70 (95%CI:0.68–0.73) of cancer-specific death in the study population, which was significantly higher than that of the classical TNM stage (C-index = 0.56, 95%CI: 0.52–0.60). The calibration plot for the probability of 1-year and 2-year survival of NSCLC patients with malignant pericardial effusion illustrated an acceptable agreement between the nomogram prediction and the actual observation (S1 Fig). A total of 348 patients were randomly assigned as the internal validated population. The C-index of internal validated population was 0.72 (95%CI: 0.69–0.76) of cancer-specific death. Therefore, this prognostic nomogram was supposed to predict whether the patients have a poor outcome in terms of cancer -specific death after two years.

### Propensity score matching of chemotherapy in the study population

In the study population, chemotherapy was regarded as the independent prognostic factor of cancer-specific death and had the largest contribution for the survival of NSCLC patients with malignant pericardial effusion. However, some variables had significant differences between the patients with chemotherapy and the patients without chemotherapy in the study population, including age, contralateral or bilateral pleural effusion, marital status and AJCC N (S1 Table). To better evaluate the impact of chemotherapy, propensity score matching was carried out to minimize the differences of these variables between two groups. After 1:1 propensity score matching, 139 paired NSCLC patients with nearly balanced variables were found (S1 Table).

**Fig 3:**
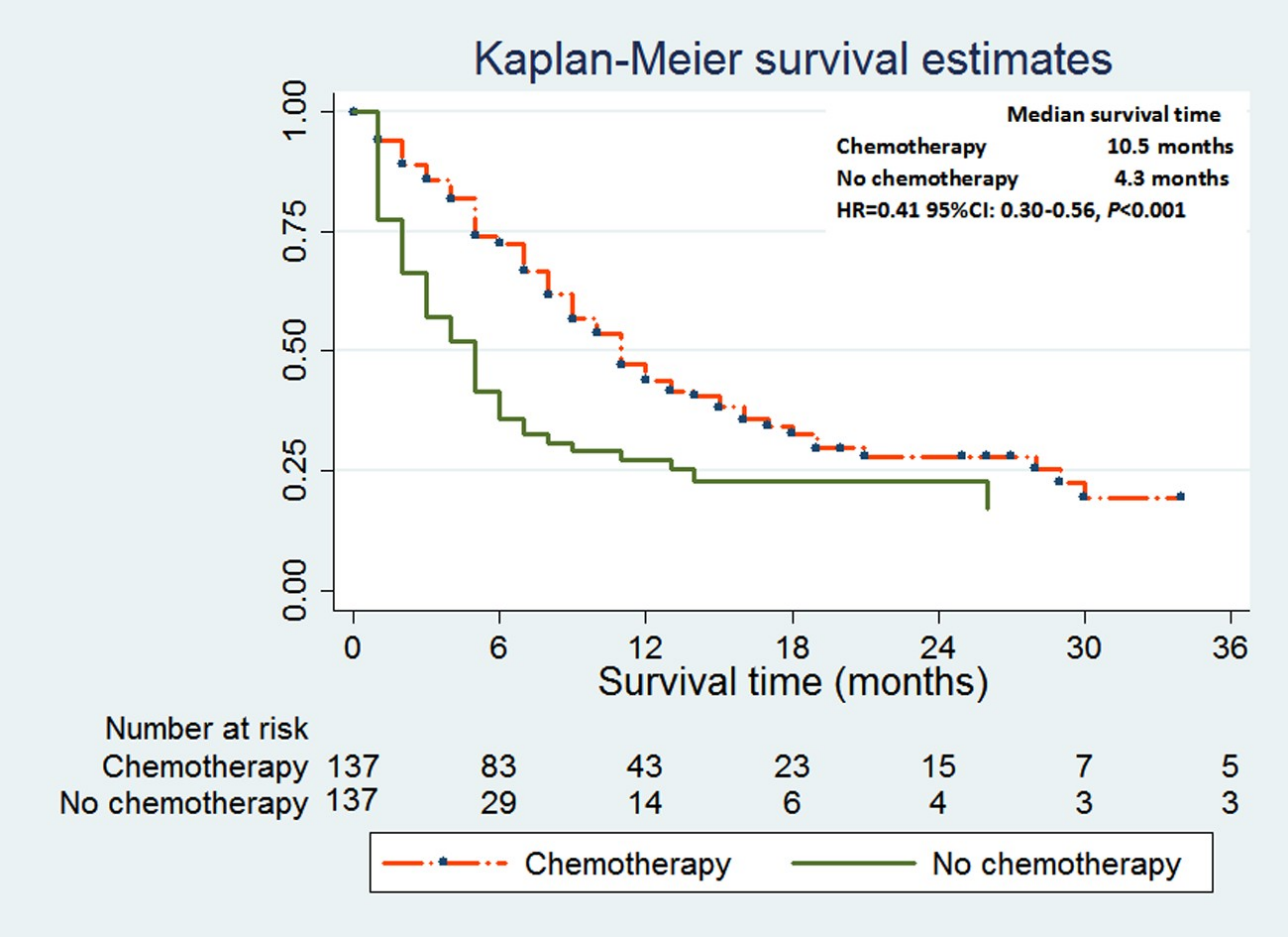
Cause-specific death in lung cancer patients with malignant pericardial effusion with or without chemotherapy after 1:1 propensity score matching.

After propensity score matching, the patients with chemotherapy had significantly longer median survival time than the patients without chemotherapy (10.5 months vs 4.3 months). There were 30.9% of 1-year survival rate and 10.8% of 2-year rate in the treatment group, compared with 10.7% of 1-year survival rate and 2.9% of 2-year rate in the control group. Chemotherapy seemed to be associated with the low risk of cancer-specific death (HR = 0.41 95%CI: 0.30–0.56, *P*<0.001) of NSCLC with malignant pericardial effusion (Fig 3).

## Discussions

Approximately 3% of lung cancer patients were associated with malignant pericardial effusion, leading to poor prognosis and many discomforts [10]. Most present studies evaluated the prognostic factors of malignant pericardial effusion secondary to multivariate tumors, mainly including lung cancer, breast cancer, hematologic malignant and gastrointestinal cancer [3,6,9]. To our best knowledge, this work is currently the largest study to evaluate prognostic factors and develop a prognostic nomogram of NSCLC alone with malignant pericardial effusion. In our study, the median survival time of the study population was 6 months, which was longer than that of the previous studies.The diagnostic year of our patients was 2010–2014. The survival time of lung cancer was gradually prolonged with the development of lung cancer treatment. The 1-year survival rate of the study population was 22.7%, which was similar to the previous results [10]. We also reported 8.9% of the 2-year survival rate.We found that laterality, AJCC N, AJCC T, and chemotherapy were the main determinants of survival time for NSCLC patients with malignant pericardial effusion through the multivariate cox regression analysis and competing risk analysis.

Chemotherapy was recognized as the main choice in treating stage IV lung cancer. However, it did not attract enough attention because of the short survival time of lung cancer with malignant pericardial effusion. Some clinical trials evaluated the effect of intrapericardial chemotherapy for malignant pericardial effusion by using multivariate antineoplastic drugs [18]. Compared with pericardiocentesis alone, intrapericardial chemotherapy combined with pericardiocentesis led to better prognosis and lower recurrence rate [16,18].Clinicians have gradually been paying the attention to systemic chemotherapy in treating lung cancer with malignant pericardial effusion. The results of chemotherapy in treating neoplastic pericardial effusion were inconsistent. Kaira and his colleagues [14] thought that systemic chemotherapy did not effectively control malignant pericardial effusion caused by lung cancer. Celik and his colleagues [15] deemed that systemic chemotherapy may be helpful to treat malignant pericardial effusion secondary to breast cancer and lymphomas, but had very minimal effect in treating lung cancer patients with malignant pericardial effusion.The study from Wang and his colleagues [19] demonstrated that NSCLC patients with cardiac tamponade who received systemic chemotherapy had better overall survival than those receiving local therapy and those receiving supportive treatment. Lestuzzi and his colleagues [16] performed a multicenter study to assess the effect of chemotherapy on 119 lung cancer patients with malignant pericardial effusion. Intrapericardial chemotherapy combined with systemic chemotherapy significantly prolonged the survival time and obtained better control of pericardial effusion compared with other treatment methods. The patients with systemic chemotherapy seemingly had the longer survival time than that without chemotherapy, although not statistically significant. However, Some limitations were also observed in this study [16]. Firstly, the treatment options were based on clinical presentation, as judged by the oncologists, and changed over time, following evolving knowledge and experience. Secondly, the patients received different therapeutic methods during the 21-year recruitment period. Over the past decade, many new drugs have been introduced into lung cancer therapy and significantly prolonged the overall survival. The recruitment period of our study population was five years after 2010, a reputation potentially associated with better overall survival compared with the above-mentioned studies. In our study, chemotherapy was regarded as the independent prognostic factor of cancer-specific death of NSCLC with pericardial effusion through the multivariate cox regression analysis(HR = 0.38, 95%CI: 0.31–0.47, *P* <0.01) and competing risk analysis(sbHR = 0.67, 95%CI: 0.53–0.86,*P* <0.01). Chemotherapy was found to share the largest contribution for the survival of NSCLC patients through the prognostic nomogram. Subsequently, the authors tried their best to decrease the selection bias through propensity score matching. We found that the patients who received chemotherapy were still associated with the longer median survival time and the lower risk of cancer-specific death than that without chemotherapy (10.5 months vs 4.3 months, HR = 0.41 95%CI: 0.30–0.56, *P*<0.01). Compared with the patients in the control group, the patients receiving chemotherapy had higher 1-year and 2-year survival rate.

Lymph node metastasis was regarded as the independent prognostic factor of lung cancer, especially mediastinal lymph node metastasis [20–22].Even in stage M1a lung cancer, lymph node involvement also had a prognostic value, along with poor outcome [23]. Mediastinal lymph node metastasis led to lymphatic obstruction and contributed to developing pericardial effusion [16]. In one study of malignant pericardial effusion secondary to multiple tumors, the patients without mediastinal lymph node enlargement had significantly longer median survival time than those with mediastinal lymph node enlargement (22.4 months vs 3.4 months, HR = 3.3; 95%CI, 1.3–8.1; *P* = 0.011) [6]. In another study of lung cancer with pericardial effusion, mediastinal lymph node metastasis was recognized as the independent factor with poor prognosis [10]. Lung cancer with malignant pericardial effusion and mediastinal lymph node metastasis was recommended to divide into stage M1b. In our study, lymph node metastasis was negatively associated with the survival of NSCLC with malignant pericardial effusion(see tables 1 and 2), which was similar to the results of previous studies. NSCLC with malignant pericardial effusion and mediastinal lymph node metastasis had significantly less median survival time of cancer-specific death (7 months vs 18 months) compared with those without lymph node metastasis in the Cox proportional hazards model (HR = 2.22, 95%CI:1.65–3.0,*P* <0.01) as well as the competing risk model (sbHR = 1.93, 95%CI:1.36–2.73, *P* = 0.01). In our study, AJCC T was also identified as an independent risk factor of cancer-specific death in NSCLC patients with malignant pericardial effusion. The patients with T1 were associated with significantly higher median survival time(14 months vs 6 months) compared with those with T4 in the Cox proportional hazards model (HR = 1.66, 95%CI:1.19–2.31, *P* <0.01) as well as the competing risk model (sbHR = 1.67, 95%CI:1.14–2.46, *P* = 0.01). Previous studies have reported that the improvement of T classification was often associated with the deteriorate of overall survival of lung cancer [24–26]. In addition, our study suggested that the patients with the left-origin of NSCLC with malignant pericardial effusion harbored worse overall survival (7 months vs 9 month) than that with the right-origin of NSCLC in the Cox proportional hazards model (HR = 0.75, 95%CI:0.61–0.92, *P* = 0.01) as well as the competing risk model (sbHR = 0.70, 95%CI:0.60–0.96, *P* = 0.02). Two recent studies showed that the right-origin lung cancer harbored lower cardiac-specific death than the left-origin lung cancer [27,28]. A potential explanation is deemed that the difference of overall survival between the left-origin lung cancer and the right-origin lung cancer can be due to asymmetries in organ size and lymphatic drainage in the mediastinum [29].

There are the following advantages in our study. Firstly, the sample of our study is currently largest, including 696 patients in the study population. All patients came from SEER database. SEER database includes cancer cases from 18 regions of the United States and covers approximate 28% of the U.S. population, which effectively avoid the selection bias of single-center study and small-sample study [30]. Secondly, we develop an prognostic nomogram for NSCLC with malignant pericardial effusion in the study population and tested its validity with good performance. The prognostic nomogram can assist clinicians to better evaluate whether the patient has a poor prognosis in term of cancer-specific death. Thirdly, we detect the impact of chemotherapy on cancer-specific death of NSCLC patients with malignant pericardial effusion through the multivariate cox regression analysis, the competing risk analysis and propensity score matching, which make our study more convincing and concrete.

Certainly, our study also had some limitations. First, the main weakness of our study is that it is unable to factor in surgical drainage into the risk equations. It has been well-documented that the primary factor determining survival of lung cancer patients with malignant pericardial effusion is decompression of the pericardium by various surgical and interventional local chemotherapy options [16,31]. Unfortunately, the SEER data base does not contain this information and so the risk equation is missing a very major element. Second, detailed information about chemotherapy was not contained in SEER database. We can not confirm that the NSCLC patients with malignant pericardial effusion receive systemic chemotherapy alone or systemic chemotherapy combined with local chemotherapy. Third, some important information in the SEER database is inadequate, such as performance status and fluid cytology of pericardial effusion, which may be the independent prognostic factor of lung cancer with pericardial effusion. Fourth, our study is a retrospective analysis, which inevitably has selection bias.

In conclusion, lung cancer with pericardial effusion has poor prognosis with approximately 8.9% of 2-year survival rate. Chemotherapy seemingly decreases the risk of cancer-specific death for NSCLC patients with pericardial effusion. AJCC N, and AJCC T are negatively associated with the survival time of NSCLC patients with pericardial effusion. The prognostic nomogram based on laterality, AJCC N, AJCC T, and chemotherapy can help to predict whether the patient is at risk of cancer-specific death within two years after the diagnosis of NSCLC with pericardial effusion.

## Supporting information

S1 Fig Calibration curve of prognostic nomogram of cancer-specific death)in non small cell lung cancer with pericardial effusion:(A) 12 months; (B) 24 months. (TIF)

S1 Table Patient characteristics group by chemotherapy and after propensity score matching. (DOCX)

## Abbreviations

HR: hazard ratio
NSCLC: non-small cell lung cancer
