# Supplementary figures and images for: Prognostic factors and nomogram for cancer-specific death in non small cell lung cancer with malignant pericardial effusion
Source: PLoS One. 2019 May 16;14(5):e0217007. doi: 10.1371/journal.pone.0217007 (PMC6521987; doi:10.1371/journal.pone.0217007)

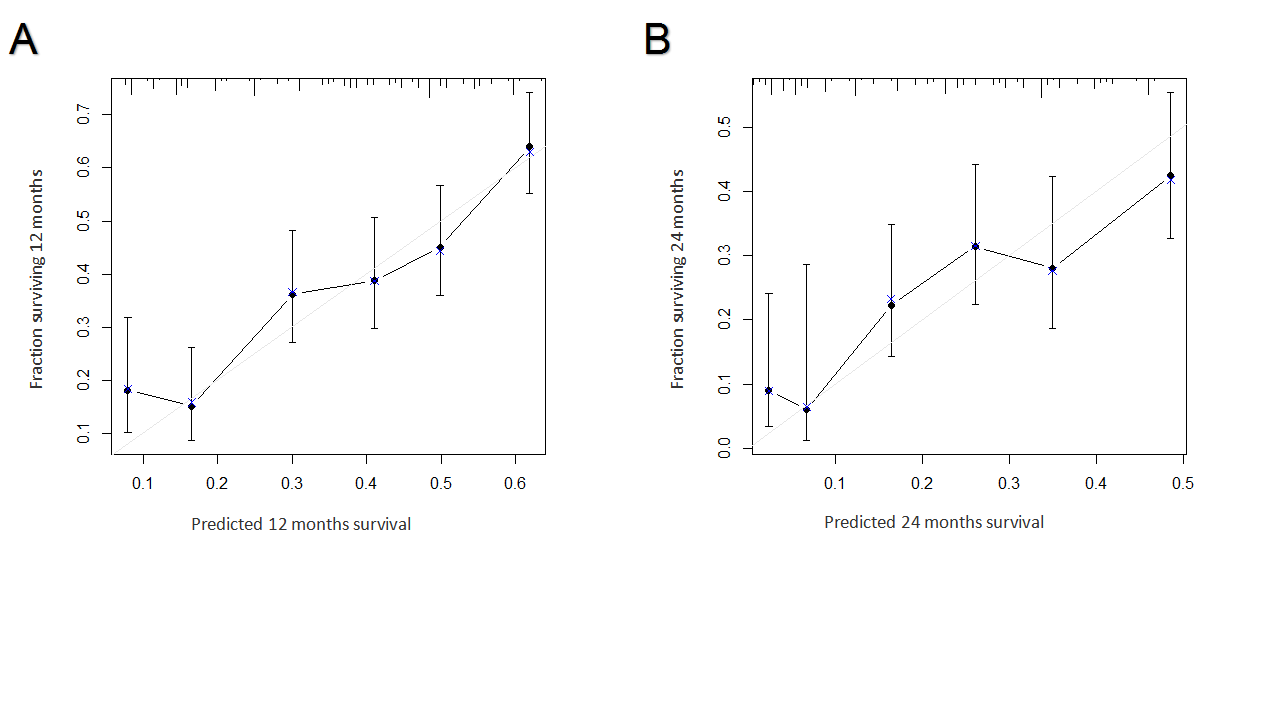

Supplement: S1 Fig — (TIF) [file pone.0217007.s001.tif]
