# Supplementary material for: Prognostic factors and nomogram for cancer-specific death in non small cell lung cancer with malignant pericardial effusion
Source: PLoS One. 2019 May 16;14(5):e0217007. doi: 10.1371/journal.pone.0217007 (PMC6521987; doi:10.1371/journal.pone.0217007)
Supplement: S1 Table — (DOCX) [file pone.0217007.s002.docx]

S1 Table: Patient characteristics group by chemotherapy and after propensity score matching

|  | | Primary data | | | Propensity score matching | | |
| --- | --- | --- | --- | --- | --- | --- | --- |
| Chemotherapy | | No | Yes | *P*-value | No | Yes | *P*-value |
| N | | 369 | 327 |  | 139 | 139 |  |
| Age(years) | | 71.5 ± 10.8 | 64.2 ± 10.6 | <0.001 | 68.1 ± 9.2 | 68.6 ± 8.6 | 0.587 |
| Sex | |  |  | 0.5 |  |  | 0.119 |
| Female | | 190 (51.5%) | 160 (48.9%) |  | 78 (56.1%) | 65 (46.8%) |  |
| Male | | 179 (48.5%) | 167 (51.1%) |  | 61 (43.9%) | 74 (53.2%) |  |
| Race | |  |  | 0.111 |  |  | 0.855 |
| White | | 295 (79.9%) | 242 (74.0%) |  | 115 (82.7%) | 112 (80.6%) |  |
| Black | | 54 (14.6%) | 56 (17.1%) |  | 15 (10.8%) | 18 (12.9%) |  |
| Other | | 20 ( 5.4%) | 29 ( 8.9%) |  | 9 ( 6.5%) | 9 ( 6.5%) |  |
| Marital status | |  |  | 0.015 |  |  | 0.28 |
| Married | | 168 (45.5%) | 179 (54.7%) |  | 66 (47.5%) | 75 (54.0%) |  |
| Unmarried | | 201 (54.5%) | 148 (45.3%) |  | 73 (52.5%) | 64 (46.0%) |  |
| Pleural effusion | |  |  | 0.016 |  |  | 0.407 |
| No | | 242 (65.6%) | 242 (74.0%) |  | 101 (72.7%) | 107 (77.0%) |  |
| Yes | | 127 (34.4%) | 85 (26.0%) |  | 38 (27.3%) | 32 (23.0%) |  |
| Primary Site | |  |  | 0.398 |  |  | 0.13 |
| Upper lobe | | 226 (61.2%) | 210 (64.2%) |  | 81 (58.3%) | 87 (62.6%) |  |
| Middle lobe | | 24 ( 6.5%) | 12 ( 3.7%) |  | 10 ( 7.2%) | 3 ( 2.2%) |  |
| Lower lobe | | 95 (25.7%) | 83 (25.4%) |  | 42 (30.2%) | 38 (27.3%) |  |
| Main bronchus | | 24 ( 6.5%) | 22 ( 6.7%) |  | 6 ( 4.3%) | 11 ( 7.9%) |  |
| Laterality | |  |  | 0.565 |  |  | 0.718 |
| Left | | 159 (43.1%) | 148 (45.3%) |  | 62 (44.6%) | 65 (46.8%) |  |
| Right | | 210 (56.9%) | 179 (54.7%) |  | 77 (55.4%) | 74 (53.2%) |  |
| Histology | |  |  | 0.46 |  |  | 0.975 |
| Squamous cell carcinoma | | 129 (35.0%) | 105 (32.1%) |  | 43 (30.9%) | 44 (31.7%) |  |
| Adenocarcinoma | | 207 (56.1%) | 198 (60.6%) |  | 83 (59.7%) | 83 (59.7%) |  |
| Other | | 33 ( 8.9%) | 24 ( 7.3%) |  | 13 ( 9.4%) | 12 ( 8.6%) |  |
| AJCC T, 7th ed | |  |  | 0.643 |  |  | 0.602 |
| T1 | | 52 (14.1%) | 46 (14.1%) |  | 16 (11.5%) | 17 (12.2%) |  |
| T2 | | 115 (31.2%) | 88 (26.9%) |  | 50 (36.0%) | 40 (28.8%) |  |
| T3 | | 88 (23.8%) | 86 (26.3%) |  | 33 (23.7%) | 40 (28.8%) |  |
| T4 | | 114 (30.9%) | 107 (32.7%) |  | 40 (28.8%) | 42 (30.2%) |  |
| AJCC N, 7th ed |  | |  | <0.001 |  |  | 0.806 |
| N0 | 104 (28.2%) | | 35 (10.7%) |  | 14 (10.1%) | 16 (11.5%) |  |
| N1 | 19 ( 5.1%) | | 23 ( 7.0%) |  | 12 ( 8.6%) | 11 ( 7.9%) |  |
| N2 | 177 (48.0%) | | 175 (53.5%) |  | 78 (56.1%) | 71 (51.1%) |  |
| N3 | 69 (18.7%) | | 94 (28.7%) |  | 35 (25.2%) | 41 (29.5%) |  |
| Tumor size(mm) | 47.6 ± 26.8 | | 50.0 ± 28.0 | 0.261 | 48.0 ± 26.0 | 50.9 ± 27.5 | 0.375 |
| Survival time  (months) | 4.8 ± 8.4 | | 11.5 ± 11.2 | <0.001 | 4.3 ± 7.7 | 10.5 ± 10.3 | <0.001 |
